# The Effect of Chronic Administration of Safranal on Systolic Blood Pressure in Rats

**Published:** 2015

**Authors:** Mohsen Imenshahidi, Bibi Marjan Razavi, Ayyoob Faal, Ali Gholampoor, Seyed Mehran Mousavi, Hossein Hosseinzadeh

**Affiliations:** a *Pharmaceutical Research Center, Department of Pharmacodynamy and Toxicology, School of Pharmacy, Mashhad University of Medical Sciences, Mashhad, Iran. *; b *Targeted Drug Delivery Research Center, Department of Pharmacodynamy and Toxicology, School of Pharmacy, Mashhad University of Medical Sciences, Mashhad, Iran. *; c *School of Pharmacy, Mashhad University of Medical Sciences, Mashhad, Iran.*

**Keywords:** *Crocus sativus*, Saffron, Safranal, Systolic blood pressure, Tail cuff

## Abstract

Safranal, the main component of *Crocus sativus* essential oil, exhibits different pharmacological activities. In this study, the effects of safranal, on blood pressure of normotensive and desoxycorticosterone acetate (DOCA) - salt induced hypertensive rats in chronic administration were investigated. Three doses of safranal (1, 2 and 4 mg/Kg/day) and spironolactone (50 mg/Kg/day) were administrated to the different groups of normotensive and hypertensive rats (at the end of 4 weeks treatment by DOCA-salt) for Five weeks. Then the effects of safranal on mean systolic blood pressure (MSBP) and heart rate (HR) were evaluated using tail cuff method. The duration of effect of safranal on SBP, was also evaluated. Our results indicated that chronic administration of safranal could reduce the MSBP in DOCA salt treated rats in a dose dependent manner. Safranal did not decrease the MSBP in normotensive rats. The data also showed that antihypertensive effects of safranal did not persist. In summary, our results showed that safranal exhibits antihypertensive and normalizing effect on BP in chronic administration.

## Introduction

*Crocus sativus* L., a member of Iridaceae family, is commonly known as saffron. It is widely cultivated in Iran and other countries (1). Safranal, the main ingradient of C. sativus essential oil, is thought to be responsible for the saffron unique odor (2). In addition, safranal is also known as a potent antioxidant (3). It was reported that safranal has anticonvulsant (4), antideppresant (5), antianxiety and hypnotic (6), antitussive (7), antinociceptive (8) and anti-ischemia (9) effects and could improve memory and learning ability in rats (10). The protective effect of safranal on diazinon induced oxidative stress has been shown recently (11). The effect of saffron and its components on cardiovascular system have also been established in some studies. It was indicated that aqueous-ethanol extract of *C. sativus,* exhibits a potent inhibitory effect on heart rate and contractility of guinea pig heart via calcium channel-blocking effect (12). Moreover, the hypotensive effect of *C. sativus* petals extract in rats has been shown previously (13). The results of our previous study revealed that the aqueous extract of saffron stigma as well as two major constitutes of this plant, crocin and safranal, has hypotensive properties in normotensive and hypertensive anaesthetized rats (14). Athough the effect of this plant in lowering blood pressure have been shown previously, but there has not been any study about the effect of safranal on blood pressure in chronic administration. Thus, in this study the effects of chronic administration of safranal, on blood pressure of normotensive and desoxycorticosterone acetate (DOCA)-salt induced hypertensive rats were investigated. 

## Experimental

*Animal and chemicals*

Adult male Wistar rats (weight 250–300 g) were provided by animal center (School of Pharmacy, Mashhad University of Medical Sciences). They were maintained on a 12 h light/dark cycle and at a temperature of 23 ± 1 °C with free access to food and water. These conditions were maintained constant throughout the experiments. The experiments were performed under the Animals (scientiﬁc procedures) Act of 1986 and conform to the National Institutes of Health guidelines for the use of experimental animals. Safranal and Tween-80 were obtained from Merck (Germany) and BDH Chemicals (Heidelberg, Germany). 5% Tween-80 was used as negative control.

*Induction of experimental hypertension*

Desoxycorticosterone acetate (DOCA)-salt (20 mg/Kg, twice weekly, for 4 weeks, s.c.) and NaCl (1%) in rat’s drinking water were used for induction of hypertension (14). Rats were randomly divided into 7 groups. 1) Saline injected (0.5 mL/Kg, twice weekly, s.c., for 4 weeks), this treatment was continued for another five weeks, 2) (DOCA)-salt (20 mg/kg, twice weekly, for 4 weeks, s.c.), DOCA treatment was continued by *i.p*. injection of 0.5 mL/Kg normal saline for another five weeks, 3, 4 and 5) (DOCA)-salt (20 mg/Kg, twice weekly, for 4 weeks, s.c.), DOCA treatment was continued by *i.p*. injection of 1, 2 and 4 mg/Kg/day safranal for another five weeks, after that safranal injection was stopped but DOCA injection was continued for another two weeks, 6) (DOCA)-salt (20 mg/Kg, twice weekly, for 4 weeks, s.c.), DOCA treatment was continued by *i.p*. injection of 50 mg/Kg/day spironolactone for another five weeks, after that spironolactone injection was stopped but DOCA injection was continued for another two weeks, 7) Saline injected (0.5 mL/Kg, twice weekly, s.c., for 4 weeks), saline treatment was continued by *i.p*. injection of 4 mg/Kg safranal for another five weeks. All groups consisted of six rats. Table 1 describes the different groups that were selected for this study. 

**Table 1:** Summary of selected groups

**Groups**	**DOCA**	**Normal saline**	**Safranal**	**Spironolactone**
1		*		
2	*	*		
3,4 and 5	*		*	
6	*			*
7		*	*	

*Hypotensive activity*

Four, nine and eleven weeks after the ﬁrst saline or DOCA treatment, SBP was measured using tail cuff method in all groups as described by Lorenz (15). Briefly, three days before the last treatment, the training of rats in different groups for indirect SBP measurements was started. This training consisted of the regular handling of the animals and getting used to the restraining cage and the tail-cuff. Rats were heated for approximately 15 minutes at 30-32 °C to increase blood flow to the tail. After that, animals were placed in small restraining cages with a cuff around the end of proximal of the tail. After placing of the cuff, a pulse transducer was used around the end of the tail. Then the tail cuff was inflated using the related button on the NIBP (Non-Invasive Blood Pressure) controller apparatus and acquisition data were performed by a computerized system Power Lab (ADInstruments, v 5.4.2). The mean values of five BPs and HRs readings were used for each animal.

*Statistical analysis*

Results are expressed as mean ± SEM. Statistical analysis was performed with ANOVA followed by Tukey–Kramer test to compare the differences between means. Differences were considered statistically significant when P<0.05.

## Results

*Effect of DOCA on SBP*

**Figure 1:**
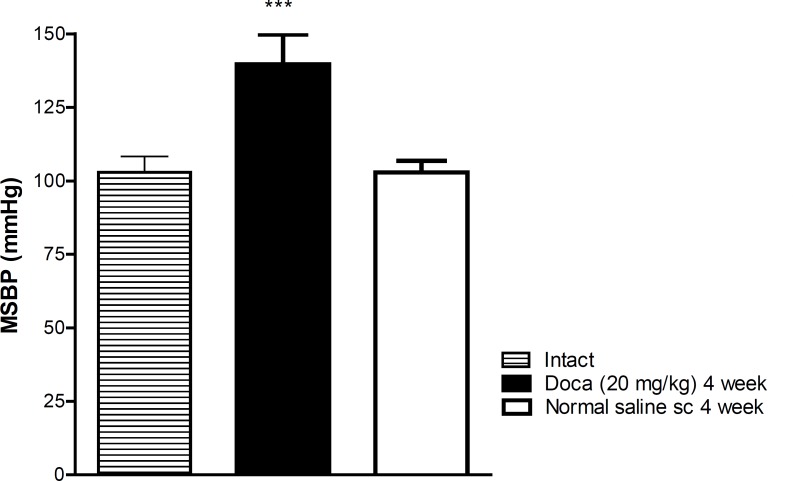
Hypertension induced by Desoxycorticosterone acetate (DOCA)-salt after 4 weeks. Each value is the mean± SEM of six experiments, *** P < 0.001 vs normal saline treated rats. One way ANOVA, Tukey Krumer test.

 In DOCA treated rats, MSBP significantly increased in comparison with normal saline treated (normotensive) rats (P < 0.001) (Figure 1).

*Effects of safranal on normotensive and hypertensive rats after nine weeks*

**Figure 2:**
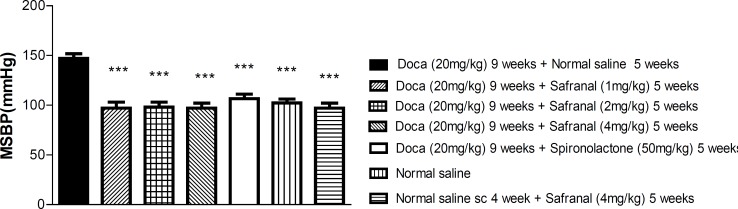
Mean systolic blood pressure (MSBP) in response to various doses of safranal in normotensive and hypertensive rats at the end of nine weeks. Each value is the mean ± SEM of six experiments. One-way ANOVA, Tukey Krumer, ***P< 0.001vs DOCA plus normal saline treated rats.

As shown in Figure 2 the injection of safranal (1, 2 and 4 mg/Kg) decreased the MSBP in hypertensive animals (P < 0.001, respectively), but in normotensive rats, safranal did not reduce the MSBP. The hypotensive effect of safranal was similar to that of spironolactone in different doses.

*Evaluation of duration effect of* *safranal on SBP*

**Figure 3:**
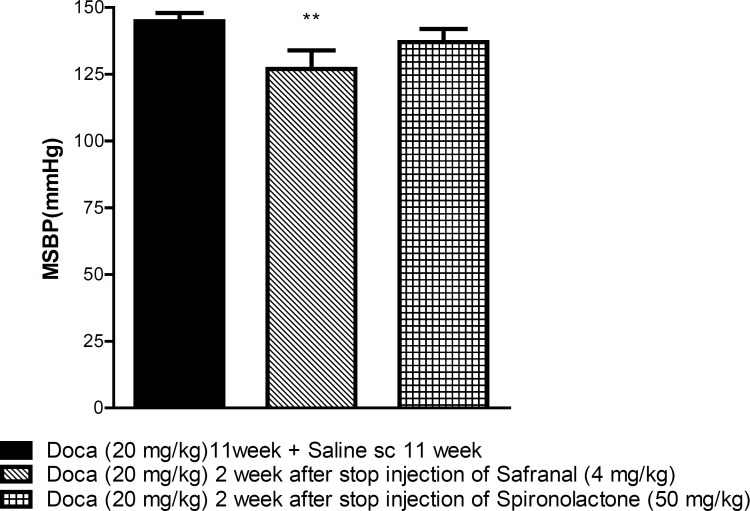
Evaluation of the duration hypotensive effect of safranal. Each value is the mean ± SEM of six experiments. One-way ANOVA, Tukey Krumer, **P< 0.001vs DOCA plus normal saline treated rats.

 As shown in Figure 3, although the hypotensive effect of safranal did not persist (97.00 ± 5.00 and 127.00 ± 7.00, before and after stopping the safranal injection, respectively), but the decrease in SBP induced by safranal, did not reach to that of DOCA treated rats after stopping the injection (P<0.01).

*Effect of safranal on heart rate*

**Figure 4:**
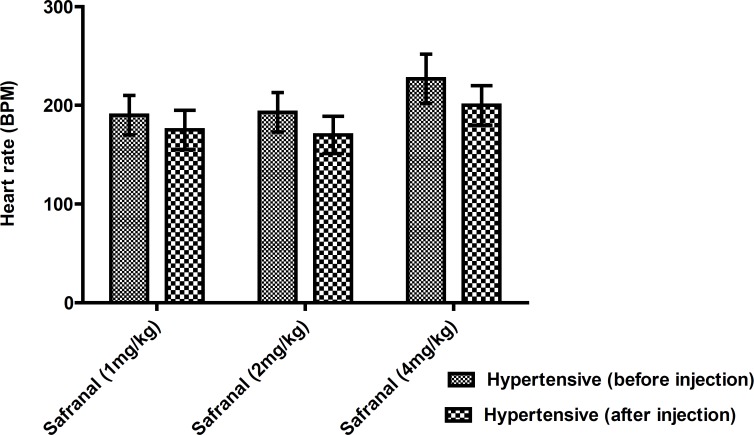
Effect of safranal on heart rate in hypertensive rats (before and after injection). Each value is the mean ± SEM of six experiments. T student test and one- way ANOVA

Safranal caused a reduction in heart rate, but the effect was not signiﬁcant (Figure 4). 

## Discussion

Deoxycorticosterone acetate (DOCA)- salt is an agent commonly used to induce hypertension in experimental animals (14). Our results showed that DOCA-salt significantly induced hypertension in comparison with saline group at the end of 4 weeks treatment. Chronic administration of safranal reduced the increase of MSBP induced by DOCA, but this hypotensive effect was not observed in normotensive rats. 

Previous studies revealed that saffron and its constituent’s exibit vasodilatory effects. For examples, a potent relaxant effect of safranal on smooth muscles of guinea pigs has been shown (16), so that it might be concluded that hypotensive effect of safranal in chronic treatment is attributed to the inhibitory effect on smooth muscles through blocking of calcium channel or inhibition of sarcoplasmic reticulum Ca^2+^ release into cytosol. Also, it was reported that aqueous and ethanolic extracts of saffron petals, reduced the MABP in anaesthetized rats (13). Moreover it was indicated that intravenous injection of aqueous extract of saffron stigma and two major constitutes of this plant exhibit hypotensive effects in normotensive and hypertensive anaesthetized rats and the effect of safranal on lowering blood pressure was more than crocin, so it could be suggested that safranal is more important than crocin in hypotensive properties of saffron (14). According to LD50 values, safranal was low-toxic in acute intraperitoneal route and practically non-toxic in acute oral administration in both mice and rats (17). Our results showed safranal did not cause reﬂex tachycardia, so it might be concluded that both heart function and blood vessels contractility are affected by safranal (14). 

Based on the previous study, one of the mechanisms involved in anti hypertensive effect of safranal like *Asperugo procumbens* (18) might be related to its effect on GABA (A)-benzodiazepine receptor complex (17). Because benzodiazepines, in pre-anesthetic doses, reduce blood pressure through decreasing peripheral resistance or cardiac output (18). 

It is indicated that administration of DOCA-salt causes pathophysiological and biochemical changes so that DOCA-salt hypertensive rats, could provide an animal model of oxidative and inflammatory stress in the cardiovascular system (20). Therefore, the DOCA-salt experiment can provide an appropriate model to evaluate of anti-oxidative or anti-inflammatory responses of natural or synthetic compounds on cardiovascular system. This also provides opportunities for the development of novel therapeutic agents for management of chronic cardiovascular disease (21). Therefore, it could be concluded that the antihypertensive effects of saffranal could be related partly due to their antioxidant properties (3). It is well known that DOCA induced hypertension causes an endothelial dysfunction in the isolated aortic rings as well as in the perfused mesenteric bed (22). Since safranal decreased SBP in hypertensive rats, our results probably show that the vasodilatory effect of safranal was endothelium dependent. 

Spironolactone, known as potassium-sparing diuretics, inhibits the effects of aldosterone by competing for intracellular mineralocorticoid receptors in the cortical collecting duct. This decreases the reabsorption of sodium and water, and decreasing the secretion of potassium (23). In this study, spironolactone was used as a positive control. Our results showed that the antihypertensive effect of safranal at the highest dose was as much as spironolactone at the end of nine weeks. It is likely that the hypotensive effect of safranal may be due to the saffron diuretic effect (1). 

To evaluate the duration of effects of safranal on reducing SBP, the safranal injection was stopped at the end of nine weeks but DOCA injections were continued for another two weeks. The data also revealed that although the hypotensive effect of the highest dose of safranal did not persist completely and SBP increased again after stopping the injection, but SBP did not restored to that of DOCA treated rats, this might be related to the incomplete elimination of safranal. 

## Conclusion

In summary, our results indicated that chronic administration of safranal could reduce the MSBP in DOCA salt treated rats. Therefore, safranal exhibits antihypertensive and normalizing effect on BP.
